# Primary care nurses’ awareness of and willingness to perform children’s oral health care

**DOI:** 10.1186/1472-6831-14-26

**Published:** 2014-03-26

**Authors:** Sepideh Rabiei, Simin Z Mohebbi, Reza Yazdani, Jorma I Virtanen

**Affiliations:** 1 Community Oral Health Department, Tehran University of Medical Sciences, P.O. Box 1439955991, Tehran, Iran; 2 Department of Community Dentistry, University of Oulu, P.O. Box 5281, 90014 Oulu, Finland; 3 Department of Oral Public Health, University of Helsinki, P.O. Box 41, FI-00014 Helsinki, Finland; 4 Oral and Maxillofacial Department, Oulu University Hospital, P.O. Box 21, 90029 Oulu, Finland

**Keywords:** Attitude, Children, Knowledge, Nurses, Oral health, Primary care providers

## Abstract

**Background:**

The majority of young children receive no early dental examination while attending primary health care for routine check-ups. Our aim was to study primary care nurses’ knowledge of oral health care (OHC) and their attitudes toward delivering OHC, as well as to assess their willingness to obtain OHC information.

**Methods:**

We conducted a cross-sectional survey of all primary-care nurses working in the public health centres of Tehran city. An anonymous self-administered questionnaire queried their knowledge in paediatric-, general and medicine-related areas of dentistry, providing knowledge scores for three domains. The nurses’ attitudes toward OHC and their willingness to obtain OHC information underwent evaluation with statements utilizing a five-point Likert scale. Altogether 680 nurses took part in the survey*.* The Chi-square test, *t*-test, one-way ANOVA and logistic regression model served for statistical analyses.

**Result:**

The mean score for the paediatric dentistry domain (3.6, SD: 1.5) was lower than for the medical (4.4, SD: 2.3) and dental domains (5.8, SD: 1.5). Obtaining higher scores in the paediatric (OR = 1.2) and dental (OR = 1.3) domains, and a greater willingness to receive OHC information (OR = 5.3), were associated with a positive attitude toward OHC. Nurses with a lower education (OR = 1.9) and better oral health behaviour (OR = 1.1) as well as those working in a non-affluent region (OR = 1.6) had a more positive attitude toward OHC.

**Conclusion:**

Primary care nurses’ low level of knowledge in OHC and their positive attitude and willingness to obtain more information point to the need for appropriate OHC training and encouragement for the nurses to promote oral health and prevent dental diseases.

## Background

The most common chronic disease affecting children’s health all over the world is dental caries, which continues to pose a serious problem, particularly in low-income populations [1,2]. Since dental care delivered by dentists is expensive, the dental caries of poor children often go untreated which leads to lower quality of life for them than for children in higher-income families [3,4]. Moreover, the majority of young children in many countries receive no dental examinations before the age of three though they frequently visit primary health care providers for routine check-ups [5,6]. These routine check-ups provide a context within to integrate oral health promotion into the practices of non-dental staff in public health centres [7,8].

Sharing the responsibility for children’s oral health care (OHC) with primary health care creates opportunities for joint ventures in target populations, since the same population is at high risk for other health and social problems. It is therefore more efficient to integrate oral health advices into general health messages by taking advantage of an approach that takes into consideration common health determinants for preventive measures [9]. This common risk factor approach calls for multi-professional collaboration [10,11]. Comprehensive approaches are certainly even more important and efficient in health care service systems with less developed public dental services.

Primary health nurses are easily accessible, as are low-cost health workers, who are in frequent contact with waiting mothers and children. Integrating OHC into these staff duties is expected to generate cost-effective preventive and health-promoting activities [12]. For example, involving vaccination staff in public health centres to deliver oral health instructions to parents has proved successful in reducing caries in toddlers [13-15]. On the other hand, studies have shown that nurses receive limited training in OHC, and their knowledge in this field is inadequate [1,16]. In addition, other studies have identified attitudinal barriers among health staff as a major challenge [17].

In this survey, we aimed to study the knowledge of and attitudes towards oral health care among primary care nurses in Tehran as well as to assess their willingness to obtain OHC information.

## Methods

### Study design and target population

We designed a cross-sectional survey by means of a self-administered questionnaire which the Ethics Committee of the Tehran University of Medical Sciences approved. The target population comprised all nurses working in the public health centres of Tehran in April 2011. Tehran has seven District Health Centres (DHC), which supervise 15 to 30 public health centres with varying numbers of nurses in each unit [18].

The DHCs agreed to distribute the questionnaires to nurses with a gift pack of a tooth-brush and dental floss and collect them in one week. The survey was voluntary and the responses were anonymous. Of the 690 nurses, 680 (97% females) returned completed questionnaires (response rate: 99%).

### Variables of the questionnaire and pilot study

We developed the study questionnaire based on previously validated surveys of nurses’ knowledge of, willingness to adopt and attitude toward OHC [18-21]. The content validity of the questionnaire was evaluated by experts in dental public health. Then we conducted a pilot study (test/re-test at a two-week interval) among physicians and nurses (n = 30) working in the public health centers of Qazvin, a city near Tehran. We revised the questionnaire according to the pilot study. The questionnaire comprised statements on knowledge, attitude, and willingness for OHC education and background factors.

### Knowledge questions

The questionnaire was a slightly modified version of one used in a similar study performed among primary care physicians and consisted of three domains: a) paediatric dental knowledge, b) general dental knowledge and c) dentistry-related medical knowledge [18].

Each domain included nine questions. In the paediatric domain, the questionnaire enquired about the time of tooth eruption; the time to start brushing and cleaning teeth and using fluoride; the transmission of bacteria; carcinogenicity of breast milk compared to the formula; and the effects of dummy sucking. The dental domain included questions on the first signs of tooth decay and its aetiology, the effects of fluoride and xylitol, the best time to refer a pregnant woman for a dental procedure, and the main cause of periodontal diseases. The medical domain consisted of questions on the relationship between systemic and periodontal diseases, and how drugs increase the risk for dental caries [18].

### Attitude questions

We measured nurses’ attitudes on a five-point Likert scale using eight statements describing the nurses’ opinions about OHC and their role in preventing oral diseases: “Dental caries and periodontitis can be stopped”, “Nurses should examine the oral cavity ”, “Routine dental visits are effective in preventing dental diseases”, “Nurses may play an important role in preventing oral diseases”, “Oral health care delivered by nurses is inefficient”, “Having oral health problems can lead to general health problems”, “I would like to implement preventive oral health activities” and “Prevention is more important than other activities”. Their responses ranged from “strongly agree” to “strongly disagree”, including “don’t know”.

### Willingness question

We also measured nurses’ willingness to obtain more training in oral health with a statement asking “How much are you willing to obtain more information about oral health?” and five Likert response alternatives: 1) very much, 2) much, 3) little, 4) very little, 5) not at all.

### Backgrounds

Socio-demographics included age, gender, educational degree, work experience (in years), and the socio-economic status of the region in which the public health centres are located (affluent/non-affluent). We also assessed the nurses’ oral health behaviour (OHB) with five questions about their “frequency of tooth brushing” (Irregularly or never, Once a week, A few (2–3) times a week, Once a day, More than once a day), “using fluoride tooth-paste” (Always or almost always, Rather often, Seldom, Never), “flossing” (Not at all, Occasionally, A few (2–3) times a week, Once a day, More than once a day), “eating sugar-containing snacks between meals” (About three times a day or more, About twice a day, About once a day, Occasionally (not every day), Rarely) and “the time of your last dental check-up” (Within six months, Six months to one year ago, One to two years ago, Two to five years ago, More than five years ago, Never, Do not remember).

### Data analyses

We dichotomised the answers to the knowledge questions to “1” for correct answers and “0” for false and “don’t know” answers. Thereafter, we calculated a total score for the knowledge category (range: 0–23) as well as its three domains [paediatric dental (range: 0–9), general dental (range: 0–9) and medical (range: 0–9)]. For the attitude questions, we scored response alternatives from 0 to 4. We then calculated a total score for each person (range: 0–32).

We coded the responses to the OHB questions such that higher scores represented more desirable habits (1–7), and then calculated a sum variable for OHB. For further analysis, we divided the sum variable of paediatric knowledge, attitude, and OHB into three categories, based on the 33rd percentile for the responses, as low, medium and high scores.

### Statistical analysis

Evaluation of the statistical significance of the differences between the subgroups included the independent samples *t*-test and one-way ANOVA for the comparison of mean values, and the Chi-square test for frequencies. We used analysis of variance with repeated measurements and the paired samples *t*-test to evaluate statistical significances between the mean scores.

A logistic regression model served in the multivariate assessment of factors related to a high score in attitude compared to those related to a low score. We then determined the corresponding odds ratios (OR) and their 95% confidence intervals (95% CI). We used the Hosmer and Lemeshow test to assess goodness of fit (p = 0.146).

## Results

### Backgrounds

The participants varied in age from 22 to 56 years (mean: 37 years, SD: 8), and a majority of them were women (97%). Table 1 shows the distribution of those primary care nurses working in the public health centres according to their background characteristics. Among the nurses, 66% had higher educational degrees (BS and MS), 27% had two-years of university education, and 7% had either a diploma or less than a diploma. In the non-affluent area, more nurses held lower degrees than did nurses in the affluent area. The nurses’ OHB score ranged from 10 to 26 (mean = 19.71, SD: 2.55).

**Table 1:** Distribution of nurses (n = 680) working in public health centres according to background characteristics in Tehran, Iran

		**DHCs Area**			
		**Affluent (%)**	**Non-affluent (%)**	**Total (%)**	**p-value**
Age					
	22-36 year-olds	125 (40)	171 (53)	296 (46)	0.001
	37-56 year-olds	190 (60)	151 (47)	341 (54)	
	Total	315 (100)	322 (100)	637 (100)	
Gender					
	Female	324 (99)	324 (96)	648 (97)	0.005
	Male	3 (1)	15 (4)	18 (3)	
	Total	327 (100)	339 (100)	666 (100)	
Educational degree					
	Diploma and lower	7 (2)	38 (12)	45 (7)	0.000
	2 years study	95 (30)	74 (24)	169 (27)	
	BS and MS	215 (68)	201 (64)	416 (66)	
	Total	317 (100)	313 (100)	630 (100)	
OHB					
	Low	75(23)	133(40)	208(32)	0.000
	Medium	150(47)	139(42)	289(44)	
	High	95(30)	61(18)	156(24)	
	Total	320 (100)	333 (100)	653 (100)	

OHB (Oral Health Behaviour): Low = 10-18, Medium = 19-21, High = 22-26.

Statistical analyses by Chi square test.

### Knowledge of oral health

The nurses’ knowledge of oral health scores appear in Table 2 according to their backgrounds. The mean score for the paediatric dentistry domain (3.6, SD: 1.5) was lower than that for the medical (4.4, SD: 2.3) and dental (5.8, SD: 1.5) domains (p < 0.001). In the paediatric dentistry domain, the smallest number of correct answers was for dummy sucking (13%). About one-fourth of the nurses knew the correct time of tooth eruption (24%), the time to use fluoride toothpaste (26%), about the transmission of bacteria from mother to child (27%), while 80% were knowledgeable about the lesser carcinogenicity of breast milk compared to the formula. In the dental domain, the lowest percentage of correct answer was for the first sign of dental caries (48%) and the highest for the aetiology of dental caries (92%). In the medical domain, the percentage of correct answers regarding the relationship between periodontal and systemic diseases ranged between 60% and 79%, while the percentages for the effects of drugs on dental caries were 20% to 40%.

**Table 2:** The nurses’ (n = 680) knowledge scores (mean, SD) in three domains according to their background characteristics in Tehran, Iran

	**Paediatric knowledge**	**Dental knowledge**	**Medical knowledge**	**Total knowledge**
	**Mean (SD)**	**Mean (SD)**	**Mean (SD)**	**Mean (SD)**
**Age**				
22-36-year-olds	3.6 (1.5)	5.8 (1.5)	4.3 (2.2)	13.8 (3.6)
37-56-year-olds	3.5 (1.4)	5.8 (1.3)	4.4 (2.3)	13.8 (3.4)
p-value	.448	.973	.565	. 973
**Gender**				
Female	3.6 (1.5)	5.8 (1.4)	4.4 (2.3)	13.7 (3.5)
Male	3.9 (1.8)	5.9 (1.5)	3.8 (2.2)	13.7 (3.7)
p-value	. 387	.597	.291	.993
**Working Area**				
Affluent	3.6 (1.5)	5.8 (1.3)	4.6 (2.3)	14.0 (3.6)
Non-Affluent	3.6 (1.5)	5.7 (1.6)	4.2 (2.2)	13.5 (3.5)
p-value	. 556	.186	.018	.067
**Work experience**				
0-5 years	3.7 (1.5)	5.8 (1.6)	4.5 (2.2)	13.9 (3.9)
6-14 years	3.4 (1.5)	5.7 (1.5)	4.3 (2.4)	13.4 (3.5)
15-30 years	3.7 (1.4)	5.9 (1.2)	4.5 (2.3)	14.1 (3.2)
p-value*	.116	.246	. 656	.119
**Educational degree**				
Diploma and lower	4.4 (1.8)	5.8 (1.3)	3.4 (2.4)	13.6 (3.4)
2 years study	3.6 (1.4)	5.8 (1.4)	4.5 (2.4)	13.9 (3.4)
BS and MS	3.4 (1.4)	5.7 (1.5)	4.5 (2.2)	13.6 (3.6)
p-value*	.000	.747	.012	.664
**OHB**				
Low	3.7 (1.6)	5.6 (1.5)	4.0 (2.3)	13.4 (3.4)
Medium	3.6 (1.5)	5.9 (1.4)	4.6 (2.10	14.0 (3.4)
High	3.5 (1.4)	5.8 (1.4)	4.6 (2.4)	13.8 (3.8)
P-value*	. 324	.215	.013	.140
**Total**£	3.6 (1.5)	5.8 (1.5)	4.4 (2.3)	13.7 (3.5)

OHB (Oral Health Behaviour): Low = 10-18, Medium = 19-21, High = 22-26.

Statistical analyses by *T*-test, *One-way ANOVA and £ Repeated measurements.

The nurses working in the affluent area had higher scores in total knowledge and in the medical domain than did their colleagues working in the non-affluent area. The knowledge of medicine score was the lowest among nurses with low OHB scores (p < 0.05).

### Attitude toward OHC

**Figure 1:**
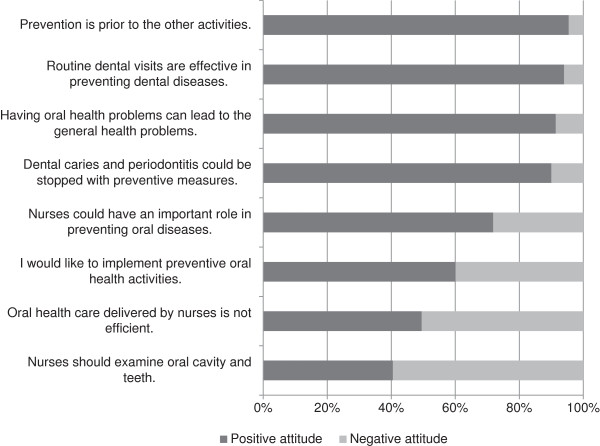
Nurses’ (n = 680) attitudes toward oral health care in public health centres in Tehran, Iran.

The mean score of the nurses’ attitudes toward OHC was 22 (SD: 5.5). Figure 1 shows that almost all nurses had generally positive attitudes toward preventive OHC. In their opinion, routine dental visits are effective (94%), and they were aware of the relationship between dental and general health (91%). Most (72%) of them believed that they could play an important role in preventing oral diseases, while 60% were interested in implementing preventive activities for their patients. Half (49%) of the nurses believed that OHC delivered by nurses is efficient, and 40% thought that they should examine the oral cavity and teeth during routine patient visits. Table 3 shows that nurses with higher paediatric knowledge scores held more positive attitudes toward preventive OHC than did those with lower paediatric knowledge scores.

**Table 3:** Primary care nurses’ (n = 680) attitudes toward dental health care and their paediatric knowledge scores in Tehran, Iran

		**Paediatric knowledge score**				
		**Low (%)**	**Medium (%)**	**High (%)**	**Total (%)**	**p-value**
**Attitude**						
	**Low**	60	115	50	225	.052
		(35)	(34)	(30)	(33)	
	**Medium**	67	118	51	236	
		(40)	(34)	(30)	(35)	
	**High**	48	109	68	219	
		(23)	(32)	(40)	(32)	
	**Total**	169	342	169	680	
		(100)	(100)	(100)	(100)	

Paediatric knowledge score: Low = 0-2, Medium = 3-4, High = 5-8.

Attitude score: Low = 0-19, Medium = 20-24, High = 25-32.

Statistical analyses by Chi square test.

### Willingness to obtain OHC information

Most nurses (69%) were willing to learn more about OHC. Younger nurses, nurses working in the non-affluent area, and those with less work experience were more willing in this regard than were their older colleagues, those who were working in the affluent area and those who had more experience (p = 0.001).

### Multivariate analysis

After controlling for other background characteristics, the logistic regression model revealed that higher scores in the paediatric (OR = 1.2, 95% CI: 1.0-1.4) and dental (OR = 1.3, 95% CI: 1.1-1.6) domains and a greater willingness to learn more about OHC (OR = 5.3, 95% CI: 3.2-8.8) were associated with a positive attitude toward OHC (Table 4).

**Table 4:** Factors associated with nurses’ (n = 680) attitudes (high/low) toward oral health care controlling for background characteristics as shown by a logistic regression model

	**B**	**S.E.**	**Sig.**	**OR**	**95% C.I. for OR**	
					**Lower**	**Upper**
Paediatric score	0.19	0.08	.020	1.21	1.03	1.43
Dental score	0.28	0.09	.001	1.33	1.12	1.57
Medical score	0.07	0.05	.170	1.08	0.97	1.19
OHB	0.11	0.05	.020	1.12	1.02	1.23
Working area	0.47	0.24	.049	1.59	1.00	2.53
Willingness to Receive information	1.67	0.26	.000	5.31	3.21	8.78
Educational degree	0.64	0.19	.001	1.89	1.29	2.77

All variables were used in their continuous form except for degree, working area, and willingness to receive information.

Educational degree: 1 = BS and MS, 2 = two years university studies, 3 = diploma and lower.

Working area: 1 = affluent area, 2 = non-affluent area.

Willingness to receive information: 0: No, 1: Yes.

Goodness of fit with Hosmer and Lemeshow test, P- value = 0.146.

Nurses with a lower educational degree (OR = 1.9, 95% CI: 1.3-2.8), better OHB (OR = 1.1, 95% CI: 1.0-1.2), and those working in the non-affluent area (OR = 1.6, 95% CI: 1.0-2.5) held a more positive attitude toward OHC than did those with a higher educational degree, a lower OHB score, and those working in the affluent area (p < 0.05).

## Discussion

Our study showed a great lack of knowledge of OHC among primary care nurses working in the public health centres of Tehran. The nurses, however, clearly acknowledged the importance of prevention and showed a great willingness as well as a positive attitude towards improving their skills in this field.

### OHC knowledge

The present study revealed a low level of oral health knowledge among the nurses; because they correctly answered on average half of the questions related to oral health knowledge. Another study has reported similar findings among primary care personnel [18]. In a study of nurse practitioners’ knowledge, opinions and practice behaviours regarding oral disease and its outcomes, Wooten et al. also reported that nurses have limited knowledge of oral health [16].

In many countries with developing health care systems, most young children are not included in regular dental recall systems by dentists until they reach the age of three to six years; they do, however, see primary care providers – including nurses – up to ten times for health screenings and vaccinations before their first birthday [19]. Consequently, this professional group can play a key role in the early prevention of oral diseases in children. Although the WHO recommends integrating oral health promotion with general health promotion and provides technical and policy support for countries to achieve this goal [22], primary care providers receive limited training in this field [1,23,24], and particularly in the field of paediatric dentistry, as our study revealed. This finding is also consistent with the low levels of knowledge of paediatric OHC among primary care physicians in the same public health care setting in Tehran [18].

As the WHO emphasised, offering oral health care in developing countries within the context of primary health care programmes is important [22]. In many health care systems, primary care nurses may be the only trustworthy source of oral health preventive information from birth based on their early relationships with young children and their parents. If given appropriate training in this field, nurses can consult with parents on, for example, feeding practices, oral home care (brushing/flossing), and the use of fluorides early on in the child's life. Moreover, they could also help to diagnose caries in their early stages by referring children to dental clinics for further examination and preventive procedures or possible treatment. Studies have shown that these auxiliary workers have a considerable impact on preventive activities and are a potential target for educational interventions [25-27].

Although the nurses’ knowledge of OHC was relatively low, priming their knowledge of oral health issues and supporting their practices would benefit the promotion of oral health. Since OHC training in the basic educational curriculum of public health nurses may often be limited, there is a need to broaden their awareness and collaboration in OHC through organised training such as continuous education. Studies have shown that continuous and periodic education offers both short-term and long-term knowledge gains and could well be planned to target primary care nurses working in public health centres [28]. Several studies (e.g. Kuronen et al. 2011) have shown that such interventions proved successful among these professionals [25].

### Attitudes among nurses

The nurses generally held positive attitudes toward OHC and admitted that they should be more knowledgeable in this field. Of the nurses, more than 90% identified the relationship between dental and general health and believed that routine dental visits are effective in preventing oral diseases. However, fewer than half of them were interested in incorporating OHC into their routine patient visits, perhaps because of their hectic daily workload or, as Fulmer et al. (2012) reported, because they may think that “oral examination is not in their scope of practice” [29]. In contrast, another study reported that nurses were interested in implementing screening for their patients [16]. Despite most nurses’ belief in their essential role in preventing oral diseases, only half of them believed that their OHC would be effective. This finding may be related to the nurses’ awareness of their low level of knowledge in the field. Nurses with higher knowledge scores, especially in the paediatric and dental domains, and those more willing to learn more about oral health showed more positive attitudes. Furthermore, in our study, nurses with higher attitude scores were more willing to receive additional training in OHC, which would likely lead to improvements in their knowledge in the area. Our findings emphasise the necessity of appropriate training and encouragement for nurses working in primary care, a process which has proved effective in providing oral health promotion and disease prevention activities [19].

Surprisingly, nurses with a lower educational degree held more positive attitudes toward OHC, possibly showing that those with more education might focus their interest on more specific duties such as visiting waiting mothers for their routine check-ups, advising them about their self-care and diet, and prescribing drugs (if needed) rather than consulting about their own or their child’s OHC.

### Willingness to obtain oral health information

Nurses in the present survey were interested in obtaining more training in OHC, which is in line with the results of a previous study in which nurses agreed on the need for more information in this topic [16] as well as on the willingness of Iranian primary health care personnel to receive more OHC training [18]. The finding that younger nurses and those who had less work experience were more willing to expand their knowledge may be due to the workload of their experienced colleagues and their lack of time [30].

### Socio-economic status

Our study included DHCs from both affluent and non-affluent parts of the city of Tehran. In the affluent parts of the city, access to private dental care practice is extensive, and the people there can more easily afford these services than can those in the non-affluent regions. The nurses’ level of knowledge proved to be lower in the non-affluent areas, where children may have a greater need for dental care [31]. Nevertheless, their positive attitude and notable insight into how to deliver OHC through their routine patient care is promising. In addition, both nurses and physicians in the non–affluent area were more willing to receive training in OHC [18]. These findings may be related to their awareness of the formidable challenges poor access to care pose to better oral health [19,32]. The nurses are in frequent contact with children from disadvantaged families who may suffer from various dental diseases. Untreated dental diseases can have a substantial impact on children’s well-being in low-income populations [33,34]. Our findings call for OHC training for primary care nurses especially among non-privileged populations.

### Primary care in Tehran

The organised public health system for delivering primary health care in Iran was established in the 1970s, and OHC was integrated into the nationwide primary health care network in 1997. Several public health care centres with different health care units, such as medical, family health, maternity and vaccination units, with a number of primary care nurses, work under the supervision of DHCs. About 60% of these centres have an OHC unit with a dentist who is in charge of basic OHC services, such as restorations, scaling and extractions, mainly for 6- to 12-year-olds through a school-based national programme, as well as for pregnant and nursing mothers [5]. Because of the extremely young population of the country and the high prevalence of dental caries in children under the age of six, a public health approach which takes this young population into account is essential and could best be achieved by incorporating the great potential of non-dental staff, including nurses in primary health care [17,18].

### Integration

Previous studies have shown that primary care medical providers, and nurses in particular, can play an important role in helping individuals gain access to OHC and in introducing successful preventive measures, particularly for children at the highest risk for early childhood caries in underserved areas [32,33,35]. The WHO Global Oral Health programme emphasises that oral health is integral and essential to general health, and that any attempt to improve oral health should be integrated into other health-promoting strategies and actions [36]. Dental and paediatric associations have also underscored the importance of integrating oral health into general health for sharing the responsibility of OHC with primary care medical practitioners [8,34].

### Strengths and limitations of the study

The study included all nurses working in the public health centres of Tehran, and the high response rate speaks for the representativeness of the present study and increases its value. Our cross-sectional design permits investigation of potential associations between one’s attitude and level of knowledge; causality clearance, however, would require a longitudinal design. Surveys with a self-administered questionnaire may also have some typical shortcomings, such over- and under reporting due to social acceptance, a phenomenon that the anonymous character of this study may have minimized*.*

## Conclusions

Our study revealed a low level of knowledge in OHC among primary care nurses, who simultaneously exhibited a generally positive attitude and notable willingness to obtain more training in this field. These findings point to an essential need for appropriate training and encouragement in OHC, especially among nurses working in developing health care systems, to promote oral health and to provide suitable conditions for the prevention of dental diseases.

## Abbreviations

DHC: District Health Centre; OHC: Oral Health Care.

## Competing interest

The authors declare that they have no competing interests.

## Pre-publication history

The pre-publication history for this paper can be accessed here:

http://www.biomedcentral.com/1472-6831/14/26/prepub

## References

[B1] Lewis CW Grossman DC Domoto PK Deyo RA The role of the pediatrician in the oral health of children: a national survey Pediatrics 2000 106 e84 10.1542/peds.106.6.e84 11099627

[B2] Hallas D Shelley D Role of pediatric nurse practitioners in oral health care Acad Pediatr 2009 9 462 466 10.1016/j.acap.2009.09.009 19945081

[B3] US Government Accountability Office Oral health: dental disease is a chronic problem among low-income population [ http://www.gao.gov/cgi-bin/getrpt?GAO/HEHS-00-72]

[B4] Spielman AI Fulmer T Eisenberg ES Alfano MC Dentistry, nursing and medicine: a comparison of core competencies J Dent Educ 2005 69 1257 1271 16275689

[B5] Samadzadeh H Hessari H Naghavi M Oral Health Situation of Iranian Children Tehran, Ministry of Health and Medical Education, Oral Health Bureau 1999 Seow WK: Biological Mechanisms of Early Childhood

[B6] Wendt LK Carlsson E Hallonsten AL Birkhed D Early dental caries risk assessment and prevention in pre-school children: evaluation of a new strategy for dental care in a field study Acta Odontol Scand 2001 59 261 266 10.1080/000163501750541101 11680643

[B7] Raybound TP Wrightson AS Massey CS Smith TA Skelton J Advanced general dentistry program directors’ attitudes on physician involvement in pediatric oral health care Spec Care Dentist 2009 29 232 236 10.1111/j.1754-4505.2009.00103.x 19886934

[B8] Hale KJ American Academy of Pedatrics section on Pediatric Dentistry Oral health risk assessment timing and establishment of the dental home Pediatrics 2003 111 1113 1116 10.1542/peds.111.5.1113 12728101

[B9] Mouradian WE The face of a child: children’s oral health and dental education J Dent Educ 2001 65 821 831 11569597

[B10] WHO (World Health Organization) Global Strategy for the Prevention and Control of non Communicable Diseases 2000 Geneva: WHO

[B11] Sheiham A Watt RG The common risk factor approach: a rational basis for promoting oral health Community Dent Oral Epidemiol 2000 28 399 406 10.1034/j.1600-0528.2000.028006399.x 11106011

[B12] Jesmin S Thind A Sarma S Does team-based primary health care improve patients’ perception of outcomes? Evidence from the 2007–08 Canadian survey of experiences with primary health Health Policy 2012 105 71 83 10.1016/j.healthpol.2012.01.008 22321527

[B13] Mohebbi SZ Virtanen JI Vahid-Golpayegani M Vehkalahti MM A cluster randomized trial of effectiveness of educational intervention in primary health care on early childhood caries Caries Res 2009 43 110 118 10.1159/000209343 19321988

[B14] Ekstrand KR Kuzmina IN Kuzmina E Christiansen ME Two and a half-year outcome of caries-preventive programs offered to groups of children in the Solntsevsky district of Moscow Caries Res 2000 34 8 19 10.1159/000016564 10601779

[B15] Feldens CA Vitolo MR Drachler Mde L A randomized trial of the effectiveness of home visits in preventing early childhood caries Community Dent Oral Epidemiol 2007 35 215 223 10.1111/j.1600-0528.2006.00337.x 17518968

[B16] Wooten KT Lee J Jared H Boggess K Wilder RS Nurse Practitioners’ and certified nurse midwives’ knowledge, opinions and practice behaviors regarding periodontal disease and adverse pregnancy outcomes J Dent Hyg 2011 85 122 131 21619740

[B17] Mohebbi SZ Early Childhood Caries and a Community Trial of its Prevention in Tehran, Iran. PhD thesis, University of Helsinki, Oral Public Health Department 2008 [ http:/ethesis.helsinki.fi/julkaisut/laa/hamma/vk/mohebbi/]

[B18] Rabiei S Mohebbi SZ Patja K Virtanen JI Physicians’ knowledge of and adherence to improving oral health BMC Public Health 2012 12 855 10.1186/1471-2458-12-855 23046660 PMC3503732

[B19] dela Cruz GG Rozier RG Slade G Dental screening and referral of young children by pediatric primary care providers Pediatrics 2004 114 642 652 10.1542/peds.2004-1269 15520094

[B20] Di Giuseppe G Nobile CG Marinelli A Angelillo IF Knowledge, attitude and practices of Pediatricians regarding the prevention of oral diseases in Italy BMC Public Health 2006 6 176 10.1186/1471-2458-6-176 16822318 PMC1543635

[B21] Prakash P Lawrence HP Harvey B Maclsaac WJ Limeback H Leake JL Early childhood caries and infant oral health: Pediatricians’ knowledge, practices and training Paediatr Child Health 2006 11 151 157 19030271 10.1093/pch/11.3.151 PMC2435315

[B22] Peterson PE Challenges to improvement of oral health in the 21st century-the approach of the WHO global oral health programme Int Dent J 2004 54 329 343 15631094 10.1111/j.1875-595x.2004.tb00009.x

[B23] Curtis JW Jr Garrison RS Jr Camp MG Dentistry in medical education: result of a comprehensive survey J Med Educ 1985 60 16 20 3965719

[B24] Midwifery curriculum [ http://fnm.tums.ac.ir/85516/sec_18/p_20.aspx?lang=fa]

[B25] Kuronen R Jallinoja P Patja K Use of and attitudes toward current care guidelines among primary and secondary care nurses in Finland Clin Nurs Res 2011 20 310 325 10.1177/1054773811407765 21558484

[B26] Amemori M Virtanen J Korhonen T Kinnunen TH Murtomaa H Impact of educational intervention on implementation of tobacco counseling among oral health professionals: a cluster randomized community trial Community Dent Oral Epidemiol 2013 41 120 129 10.1111/j.1600-0528.2012.00743.x 22934678

[B27] Ford CR Foley KT Ritchie CS Sheppard K Sawyer P Swanson M Harada CN Brown CJ Creation of an interprofessional clinical experience for healthcare professions trainees in a nursing home setting Med Teach 2013 35 544 548 10.3109/0142159X.2013.787138 23631410

[B28] Marinopoulos SS Dorman T Ratanawongsa N Wilson LM Ashar BH Magaziner JL Miller RG Thomas PA Prokopowicz GP Qayyum R Bass EB Effectiveness of continuing medical education Evid Rep Technol Assess 2007 149 1 69 PMC4781050 17764217

[B29] Fulmer T Cabrera P The primary care visit: what else could be happening? Nurs Res Pract 2012 2012 720506 10.1155/2012/720506. Epub 2012 Jun 10 22720152 10.1155/2012/720506 PMC3377192

[B30] Lewis CW Cantrell DC Domoto PK Oral health in the pediatric practice setting: a survey of Washangton State pediatrics J Public Health Dent 2004 64 111 114 10.1111/j.1752-7325.2004.tb02737.x 15180081

[B31] Newacheck PW Hughes DC Hung YY Wong S Stoddard JJ The unmet health needs of America’s children Pediatrics 2000 105 989 997 10742361

[B32] Nowak AJ Casamassimo PS Using anticipatory guidance to provide early dental intervention J Am Dent Assoc 1995 126 1156 1163 10.14219/jada.archive.1995.0337 7560574

[B33] Wysen KH Hennessy PM Lieberman MI Garland TE Johnson SM Kids Get Care: integrating preventive dental and medical care using a public health case management model J Dent Educ 2004 5 522 530 15186069

[B34] Haden NK Catalanotto FA Alexander CJ Bailit H Battrell A Broussard J Jr Buchanan J Douglass CW Fox CE 3rd Glassman P Lugo RI George M Meyerowitz C Scott ER 2nd Yaple N Bresch J Gutman-Betts Z Luke GG Moss M Sinkford JC Weaver RG Valachovic RW Improving the oral health status of all Americans: roles and responsibilities of academic dental institutions: the report of the ADEA President’s Commission J Dent Educ 2003 67 563 583 12809191

[B35] Nagarajappa R Kakatkar G Sharda AJ Asawa K Ramesh G Sandesh N Infant oral health: Knowledge, attitude and practices of parents in Udaipur India. Dent Res J (Isfahan) 2013 10 659 665 PMC3858743 24348626

[B36] Petersen PE Global policy improvement of oral health in the 21st century – implications to oral health research of world Health Assembly 2007, World Health Organization Community Dent Oral Epidemiol 2009 37 1 8 10.1111/j.1600-0528.2008.00448.x 19046331

